# Defining habitat covariates in camera-trap based occupancy studies

**DOI:** 10.1038/srep17041

**Published:** 2015-11-24

**Authors:** Jürgen Niedballa, Rahel Sollmann, Azlan bin Mohamed, Johannes Bender, Andreas Wilting

**Affiliations:** 1 Leibniz Institute for Zoo and Wildlife Research, Alfred-Kowalke-Str. 17, 10315 Berlin, Germany; 2 North Carolina State University, Department of Forestry and Environmental Resources, Campus Box 8008, Raleigh, NC 27695-7646, USA

## Abstract

In species-habitat association studies, both the type and spatial scale of habitat covariates need to match the ecology of the focal species. We assessed the potential of high-resolution satellite imagery for generating habitat covariates using camera-trapping data from Sabah, Malaysian Borneo, within an occupancy framework. We tested the predictive power of covariates generated from satellite imagery at different resolutions and extents (focal patch sizes, 10–500 m around sample points) on estimates of occupancy patterns of six small to medium sized mammal species/species groups. High-resolution land cover information had considerably more model support for small, patchily distributed habitat features, whereas it had no advantage for large, homogeneous habitat features. A comparison of different focal patch sizes including remote sensing data and an *in-situ* measure showed that patches with a 50-m radius had most support for the target species. Thus, high-resolution satellite imagery proved to be particularly useful in heterogeneous landscapes, and can be used as a surrogate for certain *in-situ* measures, reducing field effort in logistically challenging environments. Additionally, remote sensed data provide more flexibility in defining appropriate spatial scales, which we show to impact estimates of wildlife-habitat associations.

Understanding the distribution and habitat associations of wildlife species is a key topic in ecology, and important for their conservation^1^. Studying wildlife habitat associations requires appropriate definition of environmental covariates at spatial scales that are relevant to the species under study^2^. A variety of approaches and methods have been developed to generate potential explanatory variables for species distribution models. These include both information collected *in-situ*, such as measurements of vegetation, disturbances or terrain collected at and around the survey locations^3^, and information based on remote sensing or airborne land cover analyses^4^,^5^,^6^.

Data from *in-situ* habitat surveys are reliable, can provide information not readily available from remote sensing (e.g. ground cover, floristic or phenological information) and can serve for ground truthing remote sensing data. These surveys can, however, be logistically challenging, costly, time-consuming and physically demanding, depending on the terrain conditions, the habitat information of interest, and the spatial scale at which data are to be collected.

Advantages of remote sensing data include extensive data coverage over large regions, also allowing for extrapolation and mapping predicted distributions, a wide spectrum of available data sets^7^ and user-friendly GIS software to extract information from data layers. Nevertheless, spatial resolution of remote sensing data is often a limiting factor for identification of smaller land cover features, with spatial resolutions ranging from 1-km resolution carbon stock data^8^, to 250-m resolution MODIS or land cover data^9^, 30-m Landsat imagery and derived data^10^ to high-resolution (<1 m) satellite imagery.

Typical satellite based imagery is further restricted to the top vegetation layer, providing no information on three-dimensional vegetation structure or features below canopy cover. Studies using high-resolution airborne LiDAR data (down to <1 m resolution and allowing for three-dimensional imaging) have overcome these problems and shown that fine-scale variations in habitat structure can influence species distributions^11^,^12^. These highly sophisticated data are, however, expensive to obtain and difficult to analyse, and thus unavailable to many wildlife studies.

Thus, both *in-situ* and remote sensing derived covariates have their advantages and disadvantages, but only few studies compared their usefulness in wildlife distribution and habitat modelling^13^.

The choice of the suitable type of covariates used is mostly governed by knowledge of or hypotheses about the ecology and life histories of species of interest. If little or nothing is known, variables characterising the environment in general terms or proxy measures can be used.

Moreover, it is well known that ecological patterns and processes are scale-dependent^14^,^15^, and an adequate definition of spatial scale is important when modelling species-habitat associations^16^,^17^.

In an ecological context, scale is the spatial (or temporal) dimension of an object or process, characterised by grain and extent^16^,^18^,^19^. Here, grain is the spatial resolution of remote sensing data (i.e., pixel size of a raster data set), and extent is characterised by focal patches of different sizes, i.e. circular areas of different radii surrounding the sampling points^20^.

Not determining the appropriate spatial scale (either grain or extent) may lead to failure to detect species habitat associations. Ideally, the definition of spatial scale is based on ecological reasoning^21^. If known, average species home range sizes or inference from related species can help in defining the appropriate spatial extent. If no information is available, various extents can be compared via model selection procedures^22^.

Here, we used camera-trapping data from Sabah, Malaysian Borneo, in an occupancy framework, one of the most common methods to study species-habitat association while accounting for imperfect species detection^23^,^24^,^25^, to 1) assess the sensitivity of occupancy models to the spatial resolution (grain size) of land cover data; and 2) investigate what focal patch size (extent) of remotely-sensed land cover information and *in-situ* habitat variables around camera-traps is most relevant to occupancy patterns of small to medium sized mammals. Our analysis aims to draw attention to scale sensitivity of model results, assess the usefulness of high-resolution land cover data, evaluate the need for *in-situ* habitat surveys, and thereby increase the efficiency and ecological relevance of future wildlife-habitat association studies.

## Methods

### Study sites

**Figure 1:**
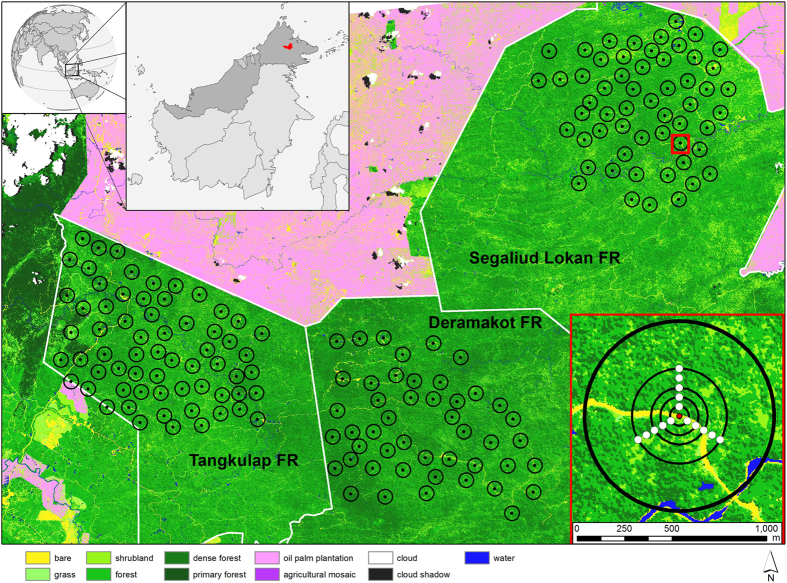
Map of the study site in Sabah, Malaysian Borneo. The Borneo map highlights the three commercial forest reserves in red. The main map shows the RapidEye land cover classification. Black dots show camera-trap locations, each with their respective 500-m radius. The bottom right inset (indicated by the red frame in the main map) magnifies one camera-trap location (central red point) with its habitat survey points along the 250-m transect lines (white points). Land cover extraction radii (extent, focal patches) are overlaid (black circles). The bold outer circle has a 500-m radius, the others correspond to 250, 150, 100 and 50 m. The 10-m radius circle is equal in size to the red point. The map was generated using ArcGIS 10.1 (ESRI, Redlands, CA, USA).

This study was conducted in three commercial forest reserves in central Sabah on Malaysian Borneo: Deramakot Forest Reserve (DFR; 551 km^2^, 5°14’-28’N, 117°20’-38’E), Tangkulap-Pinangah Forest Reserve (TFR; 501 km^2^, 5°17’-31’N, 117°03’-20’E) and Segaliud Lokan Forest Reserve (SLFR; 5°20’-39’N, 573 km^2^, 117°25’-39’E; Fig. 1). The reserves are comprised of lowland rainforest (altitude between 50–250 m) and have all been selectively logged at least once. Because of more intensive and destructive logging in the past, TFR and SLFR show higher degrees of forest disturbance than DFR, where reduced impact logging was adopted in 1995 and certification by the Forest Stewardship Council followed in 1997^3^,^26^,^27^.

### Camera-trapping

We set up 47, 64, and 55 camera-trap stations covering areas of 123 km^2^, 122 km^2^, and 114 km^2^ in DFR, TFR, and SLFR, respectively (Fig. 1). Setups approximated a systematic array with random origin, adjusted to logistical circumstances, to achieve representative coverage of the study areas. DFR was sampled between September 2008 and January 2009, TFR between April and September 2009, and SLFR between January and April 2010. Camera stations were spaced approximately 1.4 km apart; each station consisted of 2 heat-in-motion sensor triggered camera-traps (models Expert and Capture; Cuddeback, De Pere, Wisconsin) facing each other (for details, see^3^).

### Occupancy modelling

We used species detection information from camera-trapping in combination with occupancy modelling to investigate the effects of spatial resolution and extent of habitat covariates. Occupancy models use species detection/non-detection data from repeated visits to a collection of sampling sites to estimate the probability of species occurrence and its relationship with environmental covariates while accounting for imperfect species detection^23^,^24^,^25^. They consist of two components that explicitly model the ecological process (i.e. occupancy of sites) and the observation process^28^. The true occupancy state at site *i*, *z*_*i*_ (1 if present, and 0 otherwise) is considered a Bernoulli trial with probability of occupancy *Ψ*_*i*_ *: z*_*i*_ ~ Bernoulli (*Ψ*_*i*_). Since non-detection of a species at a sampling site can either be caused by true absence or by failure of detection, repeated visits over *k* occasions to sampling sites are used to estimate detection probability *p*_*ik*_ conditional on occupancy. Observations *y*_*ik*_ are also assumed to be a Bernoulli trial with *y*_*ik*_|*z*_*i*_ ~ Bernoulli (*p*_*ik*_ *z*_*i*_). Thus *p*_*ik*_ = 0 where *z*_*i*_ = 0, i.e., the species is not present.

Both occupancy probability *Ψ* at a site *i* and detection probability *p* can be modelled as linear functions of covariates *x*_*i*_ using logit link functions, e.g.:


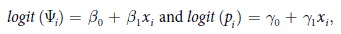


where *β*_*0*_ and *γ*_*0*_ denote the intercepts and *β*_*1*_ and *γ*_*1*_ single regression coefficients^25^. To define sampling occasions, we divided the total sampling period for each study site into 6-day sampling intervals, resulting in 7 occasions in DFR and TFR and 8 in SLFR^3^. For each species, we constructed a site-by-occasion detection/non-detection matrix with values of 1 if the species was detected at a given site on a given occasion, 0 if not and NA if the cameras were not operational.

We implemented occupancy models^25^ in R 3.1.1^29^ using package “unmarked” version 0.10-3^30^. For every species, we first selected the most suitable model for detection probability *p* using the camera position (on/off road) and forest reserve (in all combinations) as detection probability covariates while holding occupancy probability constant across sites (i.e. we used no covariates to model occupancy probability, Table S1). These models will be termed ‘constant occupancy models’ for the sake of simplicity. Model selection was based on Akaike’s Information Criterion (AIC)^31^. Conditional on the best detection model we then evaluated the effect of different covariates at varying spatial resolutions and extents on species occupancy, as described below.

We generally assessed the effects of covariates on occupancy probabilities with one occupancy covariate per model. Therefore, model rankings and inferences were not affected by correlations between related covariates.

#### Study species

We built occupancy models for six relatively small mammal species covering different taxonomic clades and ecological groups: Banded Civet *Hemigalus derbyanus* (*n* = 35 records), Long-tailed Macaque *Macaca fascicularis* (*n* = 76), Malay Civet *Viverra tangalunga* (*n* = 610), Moonrat *Echinosorex gymnura* (*n* = 140), Greater and Lesser Chevrotain *Tragulus napu and T. kanchil* (*n* = 561), and Thick-spined Porcupine *Hystrix crassispinis* (*n* = 42). As Greater and Lesser Chevrotain are difficult to distinguish with certainty on camera-trap photographs, we pooled both species and jointly analysed them.

Occupancy models assume spatial independence among sampling sites. Malay Civet^32^, Long-tailed Macaque^33^ and Chevrotains^34^ have average home range diameters smaller than our average camera-trap station spacing of 1.4 km; we assume that the same is true for the Banded Civet, Thick-spined Porcupine and Moonrat, because of their smaller size compared to the Malay Civet, and because the latter two are not carnivorous.

#### Habitat covariates

We mapped land cover using multispectral classifications of RapidEye high-resolution (5 m) satellite imagery. We used seven images (Catalog-IDs: 10606784, 10606821, 9290487, 9290518, 6890479, 10129761, 6890524) acquired between 07/2011 and 09/2012 as data base for this analysis. The RapidEye data products were supplied by the RapidEye Science Archive program (Project-ID 654) and delivered in orthorectified L3A-format^35^.

To reduce scene-to-scene variability, radiometric corrections were applied as recommended for multi-temporal and multi-sensor data applications^36^,^37^. The image-based atmospheric corrections included ‘dark object subtraction’ and conversion to exoatmospheric (top-of-atmosphere) reflectance^35^,^38^,^39^. By applying pixel-based maximum-likelihood land cover classifications, nine different land cover types were identified (Fig. 1). Clouds and cloud shadows were eliminated consulting a Landsat-based classification^40^. All images used for land cover classification were processed with ERDAS Imagine 2013 (Hexagon Geospatial, Norcross, GA, USA). The overall accuracy of the classification as estimated from 211 ground control points was 82.4%.

Based on this land cover classification we calculated four habitat covariates: distance from every camera trap station to the nearest oil palm plantation (D.PLANT) and to the nearest water pixel (D.WATER), ‘forest score’ (FS) and land cover heterogeneity (HET). The first two covariates were used to assess the sensitivity of occupancy models to spatial resolution and the latter two to test the sensitivity of occupancy models to different focal patch sizes.

FS is the weighted mean of land cover percentages within extracted areas, the weights are integer numbers assigned to each land cover class ranking forest quality. Thus, FS is an index of the degree of forest cover and quality in the surroundings of camera-traps. Bare areas, grassland, oil palm plantations and water were assigned 0, shrub 1, forest 2, dense and primary forest 3, allowing FS to range from 0 to 3. Lower numbers indicate higher disturbance of the forest.

Heterogeneity was calculated using Pielou’s evenness index, which is defined as the ratio between the actual and the highest possible Shannon diversity of members of an assemblage^41^,^42^,^43^. Values can range from 0 to 1, with 0 if a collection consists of only one class and 1 in case of perfect evenness between classes. In our context it can be interpreted as heterogeneity of land cover, because the more numerically similar the percentages of land cover classes in an area are, the more heterogeneous is land cover.

The ecological reasoning behind the choice of these covariates is that all animals depend on water to some degree and therefore access to water is a basic requirement^44^. Distance to oil palm plantations quantifies potential edge effects and can be interpreted as a proxy for human disturbance^45^. Forest score and heterogeneity are both metrics to characterise the habitat and describe the forest quality and disturbance.

In addition to habitat covariates derived from the high-resolution remote sensing data, we included one *in-situ* measured covariate into our analyses. At each camera-trap station, canopy closure (CC) was recorded every 50 m using a spherical densiometer along 3 line transects of 250 m in the direction of 0°, 120° and 240°, and the data were pooled by camera-trap station. Due to logistic constraints, not all transects could be carried out along the entire 250 m and mean effective transect length was 184 m ± 84 m. We computed CC covariates as the mean of CC values at distances of up to 50, 100 and 150 m from the camera-trap stations. 150 m was chosen as the maximum distance because 95% of all stations had at least one transect of at least that length. CC is related to forest disturbance: less disturbed forests are expected to have a more closed canopy, i.e. higher values of CC^3^.

#### Goal 1: Sensitivity of occupancy models to spatial resolution of remotely sensed land cover information

**Figure 2:**
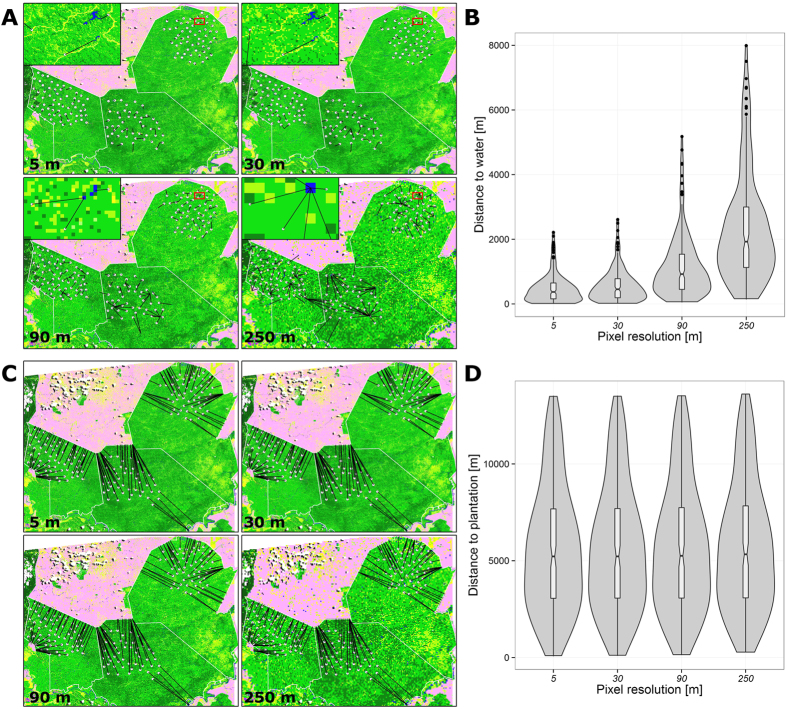
Maps and violin plots for distance to water (A,B) and distance to oil palm plantations (C,D) by pixel resolution (grain size) for three commercial forest reserves in Sabah, Malaysian Borneo. Red squares indicate position of magnified inset in A. The maps were generated using ArcGIS 10.1 (ESRI, Redlands, CA, USA).

The 5 m land cover classification was resampled to lower resolutions commonly found in other remote sensing data (30-m Landsat; 90-m ASTER; 250-m MODIS) using the majority method (i.e. by assigning each new raster cell the most common pixel value within its extent) in ArcGIS 10.1 (ESRI, Redlands, CA, USA). For all 4 resolution levels we computed the distance from every camera-trap station to the nearest oil palm plantation (D.PLANT) and to the nearest water pixel (D.WATER) (D.PLANT_5_, D.PLANT_30_ and so on, Fig. 2). The oil palm plantations represent a large continuous habitat feature, for which distances remained largely constant across spatial resolutions (Fig. 2C,D), whereas water resources were patchily distributed across our study areas and many water resources were smaller than the pixel sizes of the coarser resolutions. As a result, distance to water increased with the coarser resolutions (Fig. 2B); 30 m resolution resulted in the loss of very small ponds and streams while representing rivers well; at 90 m, most small ponds disappeared from the land cover map, medium rivers were represented in a discontinuous yet recognizable way and only large rivers were a continuous band of pixels, and at 250 m resolution even the largest river, Sungai Kinabatangan, was discontinuous, small and medium rivers and ponds mostly disappeared (Fig. 2A).

Conditional on the best constant occupancy models, we performed AIC-based model selection of occupancy covariates D.PLANT and D.WATER computed at 4 spatial resolutions for every species to assess the sensitivity of occupancy models to the spatial resolution of land cover information.

#### Goal 2: Sensitivity of occupancy models to focal patch sizes around camera-traps

We computed ‘forest score’ (FS) and land cover heterogeneity (HET) from the surroundings of the camera-trap stations using circles with radii of 10 m, 50 m, 100 m, 150 m, 250 m and 500 m (corresponding to focal patches of 0.03 ha, 0.8 ha, 3.1 ha, 7.1 ha, 16.9 ha, 78.5 ha). We chose 10 m as the minimum radius to achieve a sample size of at least 10 pixels per station, and 500 m as the maximum radius to avoid overlap between circles around neighbouring camera-trap stations. Further, we built occupancy models using the *in-situ* collected information on CC at 50, 100 and 150 m around each camera-trap.

We compared the six focal patch sizes (10 m, 50 m, 100 m, 150 m, 250 m and 500 m radii) of FS and HET land cover covariates and three focal patch sizes for *in-situ* CC measurements (50 m, 100 m, 150 m) to each other and their respective constant occupancy models using AIC-based model selection in order to find a radius at which habitat covariates had the highest predictive power for our set of species. We chose a consensus radius among those radii that were available for all covariates (50, 100, 150 m) using an ad hoc approach: We calculated the cumulative ΔAIC for each radius over all six species. A lower cumulative ΔAIC indicates that a given radius is, on average, closer to the top model than one with a higher cumulative ΔAIC.

#### Goodness of model fit

Because AIC is only a relative measure of model quality, we conducted goodness of fit tests^46^ for each species’ global model, with covariates based on the consensus radius, using the R package AICcmodavg^47^. We found no evidence for lack of fit (bootstrapped p values > 0.1 and variance inflation factors <1.5 in all global models, Supplementary Table S7) and therefore refrained from converting AIC to qAIC for model selection.

## Results

### Goal 1: Sensitivity of occupancy models to spatial resolution of land cover information

For all species, occupancy models using D.PLANT as single covariates were not influenced by the spatial resolution of land cover information. Regression coefficients and standard errors were very similar (±0.02) for spatial resolutions from 5 to 250 m; AIC values of individual models were virtually constant with ΔAIC <0.2, and AIC weights hardly differed across resolutions (see Table 1 for the Long-tailed Macaque and Supplementary Table S2 for other species).

In contrast, D.WATER models differed substantially by pixel resolution, particularly in species that exhibited strong association with this covariate. The effect was most pronounced in Long-tailed Macaque, which had a very strong (*β*_*1*_ = −3.59 ± 0.92) and highly significant negative association with distance to water at high resolution, i.e., occupancy probability *Ψ* was higher closer to water resources. AIC increased drastically and significance of regression coefficients decreased gradually with lower resolutions (Table 1). The Thick-spined Porcupine showed a significant negative association with D.WATER at all resolutions, but the effect on occupancy was strongest at 90-m resolution (see Supplementary Table S3). Only the Chevrotain showed a positive association with D.WATER, but only the coarse (250 m) resolution had more support than the constant occupancy model.

### Goal 2: Sensitivity of occupancy models to focal patch sizes around camera-traps

Generally, for FS and HET as well as CC, smaller focal patch sizes (i.e. smaller radii) had lower AIC values than larger radii in species whose occurrence was associated with the respective covariates (Table 2 for the Long-tailed Macaque and Supplementary Tables S4-S6 for other species). Particularly for the Long-tailed Macaque, the species with the strongest association with the covariates, the effect of focal patch size was pronounced, with smaller radii having more predictive power for occupancy than larger radii. Based on cumulative ΔAIC, we chose 50 m as the consensus radius for all covariates (Table 3).

## Discussion

Species habitat associations and preferences are multi-factorial processes aimed at maximizing fitness^48^ that integrate information and involve decisions made at various interacting spatial and temporal scales. To account for this complexity, analysis of habitat associations needs to be carried out at different spatial scales^2^,^49^. This is commonly achieved by analysing hierarchical selection of habitat use (e.g. at landscape, macrohabitat and microhabitat scale)^2^,^50^,^51^ or by comparing multiple focal patch sizes^20^,^17^. Both approaches aim to find relevant spatial extents, but at the same time are often influenced by the available resolution (grain size) or, in case of *in-situ* measures, availability of habitat covariates.

Our analysis showed that the effect of grain size differed by the type of land cover feature. In the case of large continuous habitat features such as oil palm plantations a higher resolution did not improve the predictive power of the covariate, as distances to these large continuous features did not depend on resolution (Fig. 2). Analogous examples to oil palm plantations would be all kinds of large land cover features that have a well-defined linear border such as other large-scale agriculture or urban areas, but also natural habitat edges or large lakes and coastlines. We expect that finer scale processes, such as movement or activity patterns, might be more sensitive to the resolution of habitat edges than the relatively coarse process of occupancy. In addition, higher resolution land cover data may have an advantage if the feature edge is less regular than in the present case.

In contrast to large land cover features, water resources were very localised, interspersed within a matrix of different land cover types and other fine-scale features in our study sites. Therefore, many of the small water resources were not visible in coarser resolution land cover data (Fig. 2B). In the set of analysed species the Long-tailed Macaque is known to be closely associated with water resources, namely rivers^52^. Our results supported the strong association with water, but we further found that even small water bodies were important for occupancy of the species, as models with a higher resolution (i.e., accounting for small water sources) had much more support than low resolution models. Very little is known about the association of the other species with water, but similar to the Long-tailed Macaque, our results showed that the Thick-spined Porcupine had a significant negative association with distance to water at all resolutions, i.e. it occurred more frequently near water. In contrast to the Long-tailed Macaque, 90-m resolution had the highest predictive power, which might indicate that this species is associated with larger rather than small water bodies. The estimated positive association of Chevrotains with distance to water was only found in the model with the lowest resolution, whereas all other resolutions led to models with less support than the constant occupancy model. If Chevrotains really avoided water, however, we would expect this pattern to be found in the higher resolutions too, similar to the results for the Thick-spined Porcupine. Therefore, we think that this finding is either an artefact of the majority method used for the resampling or a spurious relationship caused by a correlation of distance to water at coarse resolution to a habitat feature not considered in our analysis.

Analogous to localised water resources, we expect a similar effect of grain size on the predictive power of habitat covariates for other small habitat features such as individual trees in savannahs, grass patches or clearings in forests, individual houses, small-scale agriculture, burnt areas, dump sites or small roads/skid trails, i.e. features that, although present in the landscape, are not visible at coarser resolutions (i.e. at larger grain sizes), because they are smaller than one pixel.

In addition to distance-based covariates, habitat association studies often use patch characteristics around the survey locations. As ecological patterns and processes are scale-dependent^15^ adequate definition of the focal patch sizes is important^16^,^17^. Earlier studies already showed that home range size and other ecological parameters are highly important for defining the focal patch sizes and therefore, if possible, the definition of focal patch sizes should be species-specific^2^.

Often very little is known about the spatial ecology of the species of concern and therefore, species-specific focal patch sizes are difficult to define a priori. The possibility to test different focal patch sizes in order to define the adequate spatial extent for covariates and to adjust these to the spatial ecology of different species is a great advantage of remote-sensed covariates, highlighted by our study. This is particularly important in multiple-species data sets like those derived from camera-trap studies.

In this study we focused on species with small home-ranges, mainly to avoid spatial autocorrelation between camera-traps. For this set of species we found that smaller focal patches of land cover metrics and *in-situ* covariates (with a radius of 50 m) usually explained species occupancy patterns better or at least as good as larger patches. Smaller focal patches closely resemble the plots that are routinely used in vegetation assessments^40^, and it is extremely unlikely that *in-situ* covariates could be collected at focal patch sizes larger than 1 ha (corresponding approximately to the 50-m consensus radius identified in the present study) in challenging field conditions. Thus, our data indicate that for small species sampling squared plots around camera-traps would potentially have provided a more representative picture of the habitat conditions relevant for species occurrence compared to three 4-m wide 250-m long line transects and at the same time would have been easier to sample and more suitable for ground truthing remote sensing data.

Even for the species with small home ranges used in the present study, the focal patch size of 0.8 ha (corresponding to a 50-m radius around camera-trap locations) represents only a small fraction of their home-ranges^32^,^33^,^34^. We expect that for wider ranging species focal patch sizes smaller than the average home range would also have higher predictive power than home range based patches, especially in point survey based studies such as camera-trapping. We consider it unreasonable to assume that, for species with larger ranges, detections at a point can provide information about an area the size of an average home range.

The *in-situ* covariate canopy closure had high predictive power for two out of six species, but it was positively correlated with the remote sensing covariate forest score (Spearmans ρ = 0.58 and p < 0.001 between CC50 and FS50), indicating that remote sensing data can serve as a surrogate for canopy closure and potentially other *in-situ* variables. It should be noted, however, that these measures refer to different aspects of forest quality and may affect species occurrence via different mechanisms.

In addition, generating land cover classifications based on remote sensing data requires ground control points for ground truthing satellite imagery, i.e. vegetation plots that may include measurements of canopy cover or canopy closure. These plots can easily be placed around survey locations, if these represent the available land cover types. It is unnecessary, though, to perform ground truthing at all survey locations when using remote sensed covariates, consequently reducing overall field efforts.

Some habitat information, however, cannot be obtained without ground surveys. These can include human impacts like hunting (wire snares, camp sites, fire places or bullet cases) or harvesting of non-timber forest products. Proxies like distance to villages or roads derived from remote sensing data can potentially help to circumvent the need for ground based data in some circumstances, but relationships between anthropogenic impacts and remote sensed proxies need to be verified^53^. Apart from human impacts, some natural features such as salt-licks, soil types, fruiting trees or dead wood can also only be assessed on the ground. Whether *in-situ* information is needed thus depends on the research questions at hand.

Our results highlight the great potential high-resolution satellite imagery and derived landscape metrics offer for identifying species habitat associations with localised fine-scale land cover features in heterogeneous landscapes. Considering the predictive power of smaller focal patches and the advantages of high-resolution remote sensing information about certain habitat features in occupancy models, we expect that very high-resolution imagery of new satellites (grain sizes <1 m, e.g. WorldView, GeoEye or Quickbird) could further improve our ability to study species habitat associations. Even though three-dimensional and canopy structural LiDAR data^6^ have great advantages over satellite imagery, we expect that due to the high costs of LiDAR data^54^, high-resolution remote sensing data will remain the main affordable data source for many wildlife studies and may be a compromise for studying fine-scale habitat variation with low costs and relative ease of use. Such satellite data also offer the opportunity to predict species occupancy and distribution to non-sampled areas and to evaluate changes in the distribution over time in wildlife monitoring.

In summary, we showed that both spatial resolution and spatial extent of habitat covariates influence camera-trap based occupancy models. Remote sensed land cover information and derived metrics provide more flexibility than *in-situ* data to tackle these issues, and can be a surrogate for, or at least complement, the labour-intensive on-the-ground habitat assessment. This is particularly beneficial in challenging environments such as tropical rainforests, ecosystems that are known for their rich biodiversity and number of endemic, threatened and little studied vertebrate species.

## Additional Information

**How to cite this article**: Niedballa, J. *et al.* Defining habitat covariates in camera-trap based occupancy studies. *Sci. Rep.* **5**, 17041; doi: 10.1038/srep17041 (2015).

## Supplementary Material

Supplementary Information

## Figures and Tables

**Table 1:** Results of occupancy models for Long-tailed Macaque using distance to large continuous (distance to oil palm plantation) and small patchy (distance to water) remote sensed habitat features at different spatial resolutions as covariate, estimated from camera-trapping data collected between 2008 and 2010 in three commercial forest reserves in Sabah, Malaysian Borneo.

Pixel size	AIC	ΔAIC	wAIC	*β*_1_*	SE	CV	p-value**
*Distance to oil palm plantation*							
90	331.48	0	0.250	−0.72	0.29	0.4	**0.012**
5	331.5	0.02	0.247	−0.72	0.29	0.4	**0.012**
30	331.5	0.02	0.247	−0.72	0.29	0.4	**0.012**
250	331.55	0.07	0.241	−0.72	0.29	0.4	**0.012**
—	337.05	5.57	0.015	—	—	—	—
*Distance to water*							
5	301.13	0	0.997	−3.59	0.92	0.26	**<0.001**
30	312.86	11.73	0.003	−2.32	0.64	0.28	**<0.001**
90	330.35	29.22	0	−0.96	0.39	0.41	**0.014**
250	335.41	34.28	0	−0.49	0.27	0.55	0.074
—	337.05	35.92	0	—	—	—	—

ΔAIC: difference in AIC to top model, wAIC = AIC model weights, *β*_*1*_ = regression coefficient, SE = regression coefficient standard error, CV = coefficient of variation of *β*_*1*_ (SE/|*β*_*1*_|), — denotes constant occupancy model.

^*^Positive regression coefficients indicate positive association with distance to features, i.e. negative association to features. Negative regression coefficients indicate negative association with distance to features, i.e. positive association to features.

^**^Bold font indicates significance at the 0.05 level.

**Table 2:** Results of occupancy models for Long-tailed Macaque using remote sensing information and *in-situ* canopy closure at different focal patch sizes as covariates on occupancy, estimated from camera-trapping data collected between 2008 and 2010 in three commercial forest reserves in Sabah, Malaysian Borneo.

Radius	AIC	ΔAIC	wAIC	*β*_1_*	SE	CV	p-value**
*Remote Sensing – Forest Score*							
10	332.48	0	0.5	−0.68	0.27	0.4	**0.013**
50	334.61	2.13	0.173	−0.55	0.27	0.49	**0.041**
100	335.61	3.13	0.105	−0.56	0.32	0.57	0.086
150	336.04	3.56	0.084	−0.56	0.36	0.64	0.116
250	336.8	4.32	0.058	−0.52	0.39	0.75	0.185
—	337.05	4.57	0.051	—	—	—	—
500	338.1	5.62	0.03	−0.27	0.28	1.04	0.339
*Remote Sensing - Heterogeneity*							
50	329.7	0	0.455	1.03	0.38	0.37	**0.007**
10	330.87	1.17	0.253	1.02	0.42	0.41	**0.014**
100	330.92	1.22	0.247	0.83	0.32	0.39	**0.009**
150	335.66	5.96	0.023	0.5	0.28	0.56	0.075
—	337.05	7.35	0.012	—	—	—	—
250	338.33	8.63	0.006	0.22	0.26	1.18	0.402
500	338.97	9.27	0.004	0.07	0.25	3.57	0.771
*In-situ - Canopy Closure*							
50	326.53	0	0.754	−1.1	0.37	0.34	**0.003**
100	329.18	2.65	0.201	−1.07	0.42	0.39	**0.01**
150	332.33	5.8	0.042	−0.91	0.42	0.46	**0.032**
—	337.05	10.52	0.004	—	—		—

ΔAIC: difference in AIC to top model, wAIC = AIC model weights, *β*_*1*_ = regression coefficient, SE = regression coefficient standard error, CV = coefficient of variation of *β*_*1*_ (SE/|*β*_*1*_|), — denotes constant occupancy model.

^*^Positive regression coefficients indicate positive association with features. Negative regression coefficients indicate negative association with features.

^**^Bold font indicates significance at the 0.05 level.

**Table 3:** Cumulative ΔAIC for occupancy models containing covariates at different focal patch sizes (extent) across six species/species groups, estimated from camera-trapping data collected between 2008 and 2010 in three commercial forest reserves in Sabah, Malaysian Borneo.

Extent	Forest Score	Heterogeneity	Canopy closure
10	17.02	22.99	—
50	13.33	7.17	7.15
100	15.32	18.68	9.60
150	18.63	23.82	11.19
250	21.31	25.69	—
500	17.67	33.02	—

‘Extent’ refers to the radius around camera-trap stations from which covariate values were extracted. Cumulative ΔAIC was calculated for each radius over all six species. A lower cumulative ΔAIC indicates that a given radius is, on average, closer to the top model than one with a higher cumulative ΔAIC.
