# Supplementary material for: Defining habitat covariates in camera-trap based occupancy studies
Source: Sci Rep. 2015 Nov 24;5:17041. doi: 10.1038/srep17041 (PMC4657010; doi:10.1038/srep17041)
Supplement: Supplementary Information [file srep17041-s1.doc]

**Defining habitat covariates in camera-trap based occupancy studies**

Jürgen Niedballa, Rahel Sollmann, Azlan bin Mohamed, Johannes Bender, Andreas Wilting

# Supplementary Information

S 1: Best constanct occupancy models with covariates on the detection component while holding occupancy constant (based on AIC) for six mammal species/species groups, estimated from camera-trapping data collected between 2008 and 2010 in three commercial forest reserves in Sabah, Malaysian Borneo. *γ_1_* (SE) are estimates of regression coefficients for detection probability covariates with their standard errors on the logit scale; 2.5% and 97.5% CI are confidence interval bounds. Occupancy probability *ψ* is held constant within each model.

| Species | Parameter* | *γ_1_* (SE) | 2.5% CI | 97.5% CI | p-value** |
| --- | --- | --- | --- | --- | --- |
| Banded Civet | Roads  TFR/SLFR | -1.06 (0.521)  -3.22 (0.648) | -2.09  -4.49 | -0.04  -1.95 | **0.04**  **<0.001** |
| Long-tailed Macaque | Roads  SLFR | 1.18 (0.532)  -1.98 (0.631) | 0.14  -3.22 | 2.22  -0.74 | **0.02**  **0.002** |
| Malay Civet | Roads  TFR | 0.822 (0.158)  -0.797 (0.169) | 0.51  -1.13 | 1.13  -0.47 | **<0.001**  **<0.001** |
| Moonrat | TFR/SLFR | -0.66 (0.288) | -1.22 | -0.10 | **0.02** |
| Chevrotain | TFR/SLFR | -0.732 (0.158) | -1.04 | -0.42 | **<0.001** |
| Thick-spined Porcupine | Roads  TFR/SLFR | -1.046 (0.557)  -1.444 (0.668) | -2.14  -2.75 | 0.05  -0.14 | **0.06**  **0.03** |

*Roads = effect of camera-trap position on/off-road on detection probability *p* (positive coefficient signifies higher *p* on roads, negative coefficient higher *p* off roads);

TFR: Tangkulap (TFR) has different detection probability than both Segaliud Lokan (SLFR) and Deramakot (DFR)(negative coefficient signifies lower p in TFR compared to DFR and SLFR)

SLFR: SLFR has different detection probability than both TFR and DFR (negative coefficient signifies lower p in SLFR compared to TFR and DFR)

TFR/SLFR: TFR and SLFR both show a different detection probability than DFR (negative coefficient signifies lower p in TFR and SLFR as compared to DFR)

**Bold font indicates significance at the 0.05 level

S 2: Results of occupancy models using distance to oil palm plantation at different spatial resolutions as covariate; estimated from camera-trapping data on six mammal species/species groups, collected between 2008 and 2010 in three commercial forest reserves in Sabah, Malaysian Borneo. ΔAIC: relative difference in AIC to top model, wAIC = AIC model weights, *β_1_* = regression coefficient, SE = regression coefficient standard error, CV = coefficient of variation of *β_1_* (SE / |*β_1_*|), – denotes constant occupancy model.

| Species | Pixel size | AIC | ΔAIC | *w*AIC | *β_1_** | SE | CV | p-value** |
| --- | --- | --- | --- | --- | --- | --- | --- | --- |
| Banded Civet | 250 | 195.80 | 0 | 0.248 | 1.38 | 0.65 | 0.47 | **0.034** |
|  | 90 | 195.81 | 0.01 | 0.246 | 1.38 | 0.65 | 0.47 | **0.035** |
|  | 5 | 195.83 | 0.03 | 0.243 | 1.37 | 0.65 | 0.47 | **0.035** |
|  | 30 | 195.83 | 0.03 | 0.243 | 1.37 | 0.65 | 0.47 | **0.035** |
|  | - | 200.83 | 5.03 | 0.020 | - | - | - | - |
| Long-tailed Macaque | 90 | 331.48 | 0 | 0.250 | -0.72 | 0.29 | 0.4 | **0.012** |
|  | 5 | 331.50 | 0.02 | 0.247 | -0.72 | 0.29 | 0.4 | **0.012** |
|  | 30 | 331.50 | 0.02 | 0.247 | -0.72 | 0.29 | 0.4 | **0.012** |
|  | 250 | 331.55 | 0.07 | 0.241 | -0.72 | 0.29 | 0.4 | **0.012** |
|  | - | 337.05 | 5.57 | 0.015 | - | - | - | - |
| Malay Civet | - | 1344.11 | 0 | 0.383 | - | - | - | - |
|  | 250 | 1345.91 | 1.8 | 0.155 | 0.13 | 0.30 | 2.31 | 0.66 |
|  | 5 | 1345.93 | 1.82 | 0.154 | 0.13 | 0.30 | 2.31 | 0.673 |
|  | 30 | 1345.93 | 1.82 | 0.154 | 0.13 | 0.30 | 2.31 | 0.671 |
|  | 90 | 1345.94 | 1.83 | 0.153 | 0.12 | 0.30 | 2.5 | 0.681 |
| Moonrat | - | 629.62 | 0 | 0.403 | - | - | - | - |
|  | 5 | 631.61 | 1.99 | 0.149 | 0.02 | 0.18 | 9 | 0.913 |
|  | 30 | 631.61 | 1.99 | 0.149 | 0.02 | 0.18 | 9 | 0.912 |
|  | 90 | 631.61 | 1.99 | 0.149 | 0.02 | 0.18 | 9 | 0.916 |
|  | 250 | 631.61 | 1.99 | 0.149 | 0.02 | 0.18 | 9 | 0.932 |
| Chevrotain (Greater & Lesser) | 5 | 1304.20 | 0 | 0.227 | 0.38 | 0.21 | 0.55 | 0.066 |
|  | 90 | 1304.20 | 0 | 0.227 | 0.38 | 0.21 | 0.55 | 0.066 |
|  | 30 | 1304.21 | 0.01 | 0.226 | 0.38 | 0.21 | 0.55 | 0.067 |
|  | 250 | 1304.27 | 0.07 | 0.220 | 0.38 | 0.21 | 0.55 | 0.069 |
|  | - | 1305.84 | 1.64 | 0.100 | - | - | - | - |
| Thick-spined Porcupine | 30 | 250.70 | 0 | 0.231 | -0.53 | 0.30 | 0.57 | 0.079 |
|  | 5 | 250.71 | 0.01 | 0.231 | -0.53 | 0.30 | 0.57 | 0.079 |
|  | 90 | 250.80 | 0.1 | 0.220 | -0.52 | 0.30 | 0.58 | 0.083 |
|  | 250 | 250.89 | 0.19 | 0.211 | -0.51 | 0.30 | 0.59 | 0.086 |
|  | - | 252.22 | 1.52 | 0.108 | - | - | - | - |

* Positive regression coefficients indicate positive association with distance to features, i.e. negative association with features. Negative regression coefficients indicate negative association with distance to features, i.e. positive association with features.

**Bold font indicates significance at the 0.05 level

S 3: Results of occupancy model using distance to water at different spatial resolutions as covariate; estimated from camera-trapping data on six mammal species/species groups, collected between 2008 and 2010 in three commercial forest reserves in Sabah, Malaysian Borneo. ΔAIC: relative difference in AIC to top model, wAIC = AIC model weights, *β_1_* = regression coefficient, SE = regression coefficient standard error, CV = coefficient of variation of *β_1_* (SE / |*β_1_*|), – denotes constant occupancy model.

| Species | Pixel size | AIC | ΔAIC | wAIC | *β_1_** | SE | CV | p-value** |
| --- | --- | --- | --- | --- | --- | --- | --- | --- |
| Banded Civet | 90 | 198.49 | 0 | 0.532 | 0.71 | 0.38 | 0.54 | 0.062 |
|  | - | 200.83 | 2.34 | 0.165 | - | - | - | - |
|  | 30 | 201.09 | 2.6 | 0.145 | 0.44 | 0.36 | 0.82 | 0.22 |
|  | 5 | 201.87 | 3.38 | 0.098 | 0.33 | 0.36 | 1.09 | 0.354 |
|  | 250 | 202.83 | 4.34 | 0.061 | -0.03 | 0.48 | 16 | 0.948 |
| Long-tailed Macaque | 5 | 301.13 | 0 | 0.997 | -3.59 | 0.92 | 0.26 | **<0.001** |
|  | 30 | 312.86 | 11.73 | 0.003 | -2.32 | 0.64 | 0.28 | **<0.001** |
|  | 90 | 330.35 | 29.22 | 0 | -0.96 | 0.39 | 0.41 | **0.014** |
|  | 250 | 335.41 | 34.28 | 0 | -0.49 | 0.27 | 0.55 | 0.074 |
|  | - | 337.05 | 35.92 | 0 | - | - | - | - |
| Malay Civet | - | 1344.11 | 0 | 0.331 | - | - | - | - |
|  | 30 | 1344.85 | 0.74 | 0.229 | 0.32 | 0.32 | 1 | 0.309 |
|  | 5 | 1345.47 | 1.36 | 0.167 | 0.23 | 0.31 | 1.35 | 0.46 |
|  | 250 | 1345.83 | 1.72 | 0.140 | 0.15 | 0.29 | 1.93 | 0.607 |
|  | 90 | 1345.93 | 1.82 | 0.133 | 0.13 | 0.31 | 2.38 | 0.686 |
| Moonrat | - | 629.62 | 0 | 0.374 | - | - | - | - |
|  | 250 | 630.84 | 1.22 | 0.203 | -0.16 | 0.18 | 1.12 | 0.384 |
|  | 90 | 631.54 | 1.92 | 0.143 | -0.05 | 0.18 | 3.6 | 0.778 |
|  | 30 | 631.58 | 1.96 | 0.141 | 0.04 | 0.17 | 4.25 | 0.83 |
|  | 5 | 631.62 | 2 | 0.138 | 0.01 | 0.17 | 17 | 0.976 |
| Chevrotain (Greater & Lesser) | 250 | 1300.83 | 0 | 0.844 | 0.59 | 0.26 | 0.44 | **0.02** |
|  | - | 1305.84 | 5.01 | 0.069 | - | - | - | - |
|  | 90 | 1307.22 | 6.39 | 0.035 | 0.16 | 0.21 | 1.31 | 0.449 |
|  | 30 | 1307.73 | 6.9 | 0.027 | 0.06 | 0.19 | 3.17 | 0.745 |
|  | 5 | 1307.81 | 6.98 | 0.026 | 0.03 | 0.19 | 6.33 | 0.882 |
| Thick-spined Porcupine | 90 | 243.04 | 0 | 0.604 | -1.37 | 0.54 | 0.39 | **0.011** |
|  | 30 | 245.75 | 2.71 | 0.156 | -1.08 | 0.46 | 0.43 | **0.019** |
|  | 250 | 245.88 | 2.84 | 0.147 | -0.96 | 0.4 | 0.42 | **0.016** |
|  | 5 | 246.93 | 3.89 | 0.086 | -0.99 | 0.46 | 0.46 | **0.03** |
|  | - | 252.22 | 9.18 | 0.006 | - | - | - | - |

* Positive regression coefficients indicate positive association with distance to features, i.e. negative association with features. Negative regression coefficients indicate negative association with distance to features, i.e. positive association with features.

**Bold font indicates significance at the 0.05 level

S 4: Results of occupancy models using forest score (index of forest quality, ranging from 0 = bare land/oil palm plantation to 3 = dense forest) extracted for different focal patch sizes (radius) around camera traps, as covariate; estimated from camera-trapping data on six mammal species/species groups, collected between 2008 and 2010 in three commercial forest reserves in Sabah, Malaysian Borneo. ΔAIC: relative difference in AIC to top model, wAIC = AIC model weights, *β_1_* = regression coefficient, SE = regression coefficient standard error, CV = coefficient of variation of *β_1_* (SE / |*β_1_*|), – denotes constant occupancy model.

| Species | radius | AIC | ΔAIC | wAIC | *β_1_** | SE | CV | p-value** |
| --- | --- | --- | --- | --- | --- | --- | --- | --- |
| Banded Civet | 500 | 197.16 | 0 | 0.55 | -1.76 | 0.92 | 0.52 | 0.055 |
|  | 250 | 200.12 | 2.96 | 0.125 | -1.34 | 0.95 | 0.71 | 0.161 |
|  | 10 | 200.63 | 3.47 | 0.097 | 0.62 | 0.43 | 0.69 | 0.152 |
|  | - | 200.83 | 3.67 | 0.088 | - | - | - | - |
|  | 50 | 201.23 | 4.07 | 0.072 | 0.57 | 0.46 | 0.81 | 0.215 |
|  | 150 | 202.63 | 5.47 | 0.036 | -0.5 | 1.64 | 3.28 | 0.759 |
|  | 100 | 202.81 | 5.65 | 0.033 | -0.1 | 0.75 | 7.5 | 0.892 |
| Long-tailed Macaque | 10 | 332.48 | 0 | 0.5 | -0.68 | 0.27 | 0.40 | **0.013** |
|  | 50 | 334.61 | 2.13 | 0.173 | -0.55 | 0.27 | 0.49 | **0.041** |
|  | 100 | 335.61 | 3.13 | 0.105 | -0.56 | 0.32 | 0.57 | 0.086 |
|  | 150 | 336.04 | 3.56 | 0.084 | -0.56 | 0.36 | 0.64 | 0.116 |
|  | 250 | 336.80 | 4.32 | 0.058 | -0.52 | 0.39 | 0.75 | 0.185 |
|  | - | 337.05 | 4.57 | 0.051 | - | - | - | - |
|  | 500 | 338.10 | 5.62 | 0.03 | -0.27 | 0.28 | 1.04 | 0.339 |
| Malay Civet | 10 | 1339.60 | 0 | 0.552 | -0.72 | 0.32 | 0.44 | **0.024** |
|  | 50 | 1341.91 | 2.31 | 0.173 | -0.57 | 0.30 | 0.53 | 0.054 |
|  | 100 | 1343.04 | 3.44 | 0.099 | -0.5 | 0.30 | 0.60 | 0.091 |
|  | - | 1344.11 | 4.51 | 0.058 | - | - | - | - |
|  | 150 | 1344.13 | 4.53 | 0.057 | -0.41 | 0.29 | 0.71 | 0.168 |
|  | 250 | 1344.90 | 5.3 | 0.039 | -0.33 | 0.29 | 0.88 | 0.269 |
|  | 500 | 1346.03 | 6.43 | 0.022 | -0.07 | 0.26 | 3.71 | 0.773 |
| Moonrat | 100 | 626.41 | 0 | 0.322 | 0.44 | 0.20 | 0.45 | **0.029** |
|  | 50 | 626.55 | 0.14 | 0.301 | 0.43 | 0.20 | 0.47 | **0.03** |
|  | 150 | 628.22 | 1.81 | 0.13 | 0.35 | 0.20 | 0.57 | 0.076 |
|  | 10 | 628.59 | 2.18 | 0.108 | 0.32 | 0.19 | 0.59 | 0.087 |
|  | - | 629.62 | 3.21 | 0.065 | - | - | - | - |
|  | 250 | 630.59 | 4.18 | 0.04 | 0.19 | 0.19 | 1 | 0.318 |
|  | 500 | 630.95 | 4.54 | 0.033 | 0.15 | 0.18 | 1.20 | 0.414 |
| Chevrotain (Greater & Lesser) | 100 | 1297.52 | 0 | 0.309 | 0.63 | 0.21 | 0.33 | **0.003** |
|  | 150 | 1298.04 | 0.52 | 0.239 | 0.63 | 0.22 | 0.35 | **0.005** |
|  | 500 | 1298.60 | 1.08 | 0.18 | 0.62 | 0.22 | 0.35 | **0.005** |
|  | 50 | 1299.11 | 1.59 | 0.139 | 0.57 | 0.20 | 0.35 | **0.005** |
|  | 250 | 1299.36 | 1.84 | 0.123 | 0.58 | 0.22 | 0.38 | **0.008** |
|  | - | 1305.84 | 8.32 | 0.005 | - | - | - | - |
|  | 10 | 1306.09 | 8.57 | 0.004 | 0.25 | 0.19 | 0.76 | 0.189 |
| Thick-spined Porcupine | 500 | 248.96 | 0 | 0.42 | -0.77 | 0.42 | 0.55 | 0.067 |
|  | 250 | 251.67 | 2.71 | 0.109 | -0.48 | 0.38 | 0.79 | 0.204 |
|  | 150 | 251.70 | 2.74 | 0.107 | -0.45 | 0.31 | 0.69 | 0.155 |
|  | 10 | 251.76 | 2.8 | 0.104 | -0.42 | 0.28 | 0.67 | 0.125 |
|  | 50 | 252.05 | 3.09 | 0.09 | -0.4 | 0.27 | 0.68 | 0.146 |
|  | 100 | 252.06 | 3.1 | 0.089 | -0.4 | 0.28 | 0.70 | 0.159 |
|  | - | 252.22 | 3.26 | 0.082 | - | - | - | - |

* Positive regression coefficients indicate positive association with features. Negative regression coefficients indicate negative association features.

** Bold font indicates significance at the 0.05 level

S 5: Results for occupancy models using heterogeneity (index of habitat heterogeneity), extracted for different focal patch sizes (radius) around camera traps, as covariate; estimated from camera-trapping data on six mammal species/species groups, collected between 2008 and 2010 in three commercial forest reserves in Sabah, Malaysian Borneo. ΔAIC: relative difference in AIC to top model, wAIC = AIC model weights, *β_1_* = regression coefficient, SE = regression coefficient standard error, CV = coefficient of variation of *β_1_* (SE / |*β_1_*|), – denotes constant occupancy model.

| Species | radius | AIC | ΔAIC | wAIC | *β_1_** | SE | CV | p-value** |
| --- | --- | --- | --- | --- | --- | --- | --- | --- |
| Banded Civet | 50 | 198.99 | 0 | 0.393 | -0.97 | 0.61 | 0.63 | 0.112 |
|  | 500 | 200.70 | 1.71 | 0.167 | 0.52 | 0.39 | 0.75 | 0.188 |
|  | - | 200.83 | 1.84 | 0.156 | - | - | - | - |
|  | 250 | 201.69 | 2.7 | 0.102 | 0.57 | 0.78 | 1.37 | 0.465 |
|  | 100 | 202.65 | 3.66 | 0.063 | -0.23 | 0.55 | 2.39 | 0.676 |
|  | 10 | 202.73 | 3.74 | 0.061 | 0.17 | 0.53 | 3.12 | 0.745 |
|  | 150 | 202.82 | 3.83 | 0.058 | -0.05 | 0.49 | 9.8 | 0.921 |
| Long-tailed Macaque | 50 | 329.70 | 0 | 0.455 | 1.03 | 0.38 | 0.37 | **0.007** |
|  | 10 | 330.87 | 1.17 | 0.253 | 1.02 | 0.42 | 0.41 | **0.014** |
|  | 100 | 330.92 | 1.22 | 0.247 | 0.83 | 0.32 | 0.39 | **0.009** |
|  | 150 | 335.66 | 5.96 | 0.023 | 0.5 | 0.28 | 0.56 | 0.075 |
|  | - | 337.05 | 7.35 | 0.012 | - | - | - | - |
|  | 250 | 338.33 | 8.63 | 0.006 | 0.22 | 0.26 | 1.18 | 0.402 |
|  | 500 | 338.97 | 9.27 | 0.004 | 0.07 | 0.25 | 3.57 | 0.771 |
| Malay Civet | 50 | 1337.90 | 0 | 0.866 | 0.74 | 0.29 | 0.39 | **0.011** |
|  | - | 1344.11 | 6.21 | 0.039 | - | - | - | - |
|  | 10 | 1344.46 | 6.56 | 0.033 | 0.3 | 0.23 | 0.77 | 0.179 |
|  | 150 | 1345.80 | 7.9 | 0.017 | 0.16 | 0.28 | 1.75 | 0.586 |
|  | 500 | 1345.90 | 8 | 0.016 | 0.13 | 0.27 | 2.08 | 0.642 |
|  | 250 | 1346.01 | 8.11 | 0.015 | 0.09 | 0.29 | 3.22 | 0.752 |
|  | 100 | 1346.08 | 8.18 | 0.015 | 0.05 | 0.27 | 5.4 | 0.855 |
| Moonrat | 100 | 624.73 | 0 | 0.495 | -0.49 | 0.19 | 0.39 | **0.012** |
|  | 150 | 626.05 | 1.32 | 0.256 | -0.44 | 0.19 | 0.43 | **0.023** |
|  | 250 | 627.91 | 3.18 | 0.101 | -0.36 | 0.19 | 0.53 | 0.062 |
|  | 50 | 628.87 | 4.14 | 0.063 | -0.3 | 0.18 | 0.6 | 0.102 |
|  | - | 629.62 | 4.89 | 0.043 | - | - | - | - |
|  | 10 | 630.56 | 5.83 | 0.027 | 0.2 | 0.19 | 0.95 | 0.312 |
|  | 500 | 631.61 | 6.88 | 0.016 | 0.02 | 0.18 | 9 | 0.922 |
| Chevrotain (Greater & Lesser) | 250 | 1304.42 | 0 | 0.344 | -0.36 | 0.20 | 0.56 | 0.068 |
|  | - | 1305.84 | 1.42 | 0.169 | - | - | - | - |
|  | 100 | 1306.11 | 1.69 | 0.148 | -0.26 | 0.20 | 0.77 | 0.197 |
|  | 150 | 1306.76 | 2.34 | 0.107 | -0.2 | 0.20 | 1 | 0.301 |
|  | 10 | 1307.12 | 2.7 | 0.089 | -0.17 | 0.21 | 1.24 | 0.417 |
|  | 50 | 1307.45 | 3.03 | 0.075 | -0.12 | 0.19 | 1.58 | 0.539 |
|  | 500 | 1307.67 | 3.25 | 0.068 | -0.08 | 0.19 | 2.38 | 0.679 |
| Thick-spined Porcupine | 50 | 250.28 | 0 | 0.418 | -0.48 | 0.24 | 0.5 | **0.048** |
|  | - | 252.22 | 1.94 | 0.158 | - | - | - | - |
|  | 150 | 252.75 | 2.47 | 0.122 | -0.32 | 0.27 | 0.84 | 0.232 |
|  | 10 | 253.27 | 2.99 | 0.094 | -0.24 | 0.24 | 1 | 0.32 |
|  | 250 | 253.35 | 3.07 | 0.09 | -0.24 | 0.26 | 1.08 | 0.356 |
|  | 500 | 254.19 | 3.91 | 0.059 | -0.05 | 0.25 | 5 | 0.851 |
|  | 100 | 254.21 | 3.93 | 0.059 | -0.03 | 0.26 | 8.67 | 0.907 |

* Positive regression coefficients indicate positive association with features. Negative regression coefficients indicate negative association features.

*Bold font indicates significance at the 0.05 level

S 6: Results for occupancy models using canopy closure, collected in-situ along transects around camera traps and averaged across transects of different length (radius), as covariate on occupancy; estimated from camera-trapping data on six mammal species/species groups, collected between 2008 and 2010 in three commercial forest reserves in Sabah, Malaysian Borneo. ΔAIC: relative difference in AIC to top model, wAIC = AIC model weights, *β_1_* = regression coefficient, SE = regression coefficient standard error, CV = coefficient of variation of *β_1_* (SE / |*β_1_*|), – denotes constant occupancy model.

| Species | radius | AIC | ΔAIC | wAIC | *β_1_** | SE | CV | p-value** |
| --- | --- | --- | --- | --- | --- | --- | --- | --- |
| Banded Civet | - | 200.83 | 0 | 0.311 | - | - | - | - |
|  | 50 | 200.95 | 0.12 | 0.293 | 0.79 | 0.57 | 0.72 | 0.165 |
|  | 150 | 201.51 | 0.68 | 0.221 | 0.7 | 0.59 | 0.84 | 0.236 |
|  | 100 | 201.98 | 1.15 | 0.175 | 0.54 | 0.56 | 1.04 | 0.335 |
| Long-tailed Macaque | 50 | 326.53 | 0 | 0.754 | -1.1 | 0.37 | 0.34 | **0.003** |
|  | 100 | 329.18 | 2.65 | 0.201 | -1.07 | 0.42 | 0.39 | **0.01** |
|  | 150 | 332.33 | 5.8 | 0.042 | -0.91 | 0.42 | 0.46 | **0.032** |
|  | - | 337.05 | 10.52 | 0.004 | - | - | - | - |
| Malay Civet | - | 1344.11 | 0 | 0.372 | - | - | - | - |
|  | 50 | 1344.82 | 0.71 | 0.261 | -0.34 | 0.32 | 0.94 | 0.29 |
|  | 100 | 1345.44 | 1.33 | 0.192 | -0.25 | 0.32 | 1.28 | 0.438 |
|  | 150 | 1345.61 | 1.5 | 0.176 | -0.21 | 0.31 | 1.48 | 0.502 |
| Moonrat | - | 629.62 | 0 | 0.467 | - | - | - | - |
|  | 100 | 631.51 | 1.89 | 0.182 | 0.06 | 0.18 | 3 | 0.735 |
|  | 150 | 631.58 | 1.96 | 0.176 | 0.04 | 0.18 | 4.5 | 0.834 |
|  | 50 | 631.59 | 1.97 | 0.175 | -0.03 | 0.18 | 6 | 0.856 |
| Chevrotain (Greater & Lesser) | 150 | 1288.66 | 0 | 0.6 | 0.83 | 0.20 | 0.24 | **<0.001** |
|  | 100 | 1290.16 | 1.5 | 0.283 | 0.80 | 0.20 | 0.25 | **<0.001** |
|  | 50 | 1291.93 | 3.27 | 0.117 | 0.77 | 0.20 | 0.26 | **<0.001** |
|  | - | 1305.84 | 17.18 | 0 | - | - | - | - |
| Thick-spined Porcupine | - | 252.22 | 0 | 0.37 | - | - | - | - |
|  | 50 | 253.30 | 1.08 | 0.216 | -0.28 | 0.30 | 1.07 | 0.344 |
|  | 100 | 253.30 | 1.08 | 0.216 | -0.28 | 0.29 | 1.04 | 0.342 |
|  | 150 | 253.47 | 1.25 | 0.199 | -0.25 | 0.28 | 1.12 | 0.387 |

* Positive regression coefficients indicate positive association with features. Negative regression coefficients indicate negative association features.

**Bold font indicates significance at the 0.05 level

**S 7 Results of the Goodness-of-fit tests for each species’ global model as computed by the mb.gof.test function from the R package AICcmodavg with the consensus radius of 50 m for Forest Score, Heterogeneity and canopy closure and distance to water and distance to oil palm plantation computed at 5-m resolution.**

| Species | p-value* | Variance inflation factor |
| --- | --- | --- |
| Banded Civet | 0.182 | 1.3 |
| Long-tailed Macaque | 0.108 | 1.44 |
| Malay Civet | 0.112 | 1.13 |
| Moonrat | 0.104 | 1.36 |
| Chevrotain (Greater & Lesser) | 0.117 | 1.15 |
| Thick-spined Porcupine | 0.241 | 1.05 |

* p-values < 0.05 indicate lack of fit
